# Approaches in line with human physiology to prevent skin aging

**DOI:** 10.3389/fphys.2023.1279371

**Published:** 2023-10-25

**Authors:** Nazli Karimi

**Affiliations:** Department of Physiology, Faculty of Medicine, Hacettepe University, Ankara, Türkiye

**Keywords:** skin aging, prevent & control, physiology, management, innovation

## Abstract

Skin aging is a complex process that is influenced by intrinsic and extrinsic factors that impact the skin’s protective functions and overall health. As the body’s outermost layer, the skin plays a critical role in defending it against external threats, regulating body temperature, providing tactile sensation, and synthesizing vitamin D for bone health, immune function, and body homeostasis. However, as individuals age, the skin undergoes structural and functional changes, leading to impairments in these essential functions. In contemporary society, there is an increasing recognition of skin health as a significant indicator of overall wellbeing, resulting in a growing demand for anti-aging products and treatments. However, these products often have limitations in terms of safety, effective skin penetration, and potential systemic complications. To address these concerns, researchers are now focusing on approaches that are safer and better aligned with physiology of the skin. These approaches include adopting a proper diet and maintaining healthy lifestyle habits, the development of topical treatments that synchronize with the skin’s circadian rhythm, utilizing endogenous antioxidant molecules, such as melatonin and natural products like polyphenols. Moreover, exploring alternative compounds for sun protection, such as natural ultraviolet (UV)-absorbing compounds, can offer safer options for shielding the skin from harmful radiation. Researchers are currently exploring the potential of adipose-derived stem cells, cell-free blood cell secretome (BCS) and other endogenous compounds for maintaining skin health. These approaches are more secure and more effective alternatives which are in line with human physiology to tackle skin aging. By emphasizing these innovative strategies, it is possible to develop effective treatments that not only slow down the skin aging process but also align better with the natural physiology of the skin. This review will focus on recent research in this field, highlighting the potential of these treatments as being safer and more in line with the skin’s physiology in order to combat the signs of aging.

## 1 Introduction

Aging is a complex process that is characterized by a decline in physiological function and integrity. It involves macromolecular damage, impaired tissue renewal, and altered cellular signaling. This leads to tissue dysfunction, organ failure, and mortality (López-Otín et al., 2013; Zhang et al., 2015; Singh et al., 2019). Interestingly, skin aging can influence the aging phenotype in other organs. The skin’s condition has been linked to overall health, risk of mortality, and longevity. The health and appearance of the skin often serve as visible indicators of an individual’s general wellbeing (Gunn et al., 2013; Gunn et al., 2016; Huang et al., 2020). Current molecular data indicate that skin aging, especially intrinsic aging, closely reflects age-related changes seen throughout the entire human body (Nikolakis et al., 2013; Zouboulis et al., 2019). Acknowledging skin aging as a disease offers significant benefits to public health and research endeavors. Raising awareness about the strategies used to mitigate the progression of this condition, such as maintaining a healthy diet, avoiding exposure to ultraviolet (UV) light (both natural and artificial), and supporting the skin’s responsive functions, is of utmost importance. Studying skin aging goes beyond cosmetic benefits. It can provide valuable insights that can be used to develop innovative strategies for managing age-related issues in internal organs, owing to the protective role played by youthful skin (Blagosklonny, 2018). Indeed, as research on discovering more effective treatments and procedures for skin aging progresses, Safety, optimal skin penetration, and potential systemic adverse effects continue to be areas of concern. In response to these concerns, researchers have recently been moving toward approaches that prioritize safety and are better suited to the intricacies of the skin’s physiology.

In this review, I will explore cutting-edge approaches with promising potential to combat skin aging. These approaches encompass Nutritional skin care and lifestyle habits, topical treatments in harmony with the skin’s circadian rhythm (Draelos et al., 2020), endogenous antioxidant utilization, such as melatonin and natural polyphenols (Bocheva et al., 2022a), and the exploration of alternative compounds for sun protection, including naturally occurring UV-absorbing compounds (Kageyama and Waditee-Sirisattha, 2019; Alvares et al., 2022). Additionally, emerging research has been investigating the potential of adipose-derived stem cells (ADSCs) (Wang et al., 2018), cell-free blood cell secretome (BCS) (Kerscher et al., 2022), and other endogenous-like compounds (De Tollenaere et al., 2023) to promote skin health while reducing the potential risks. By emphasizing these innovative strategies, we can pave the way for a new era in skin aging interventions, one that prioritizes safety, aligns with the body’s physiological processes, and unlocks the potential for more effective and personalized anti-aging treatments.

## 2 Skin aging

### 2.1 Molecular mechanisms of aging: focusing on skin aging

As we age, our physiological functions naturally decline, and we find it increasingly challenging to maintain internal equilibrium in response to external changes. This reduced capacity to sustain homeostasis impacts our overall wellbeing (Sergiev et al., 2015). The degradation of crucial skin functions resulting from intrinsic and extrinsic aging gives rise to clinical manifestations that closely resemble several age-related internal diseases. Intrinsic (chronological) aging occurs gradually over time and affects tissues throughout the body. Extrinsic aging (photoaging), driven by environmental factors like lifestyle, nutrition, and UV radiation, accelerates the aging process and leads to visible signs of aging on the skin (Nikolakis et al., 2013; Zouboulis et al., 2019). General aging as a phenomenon is influenced by various pathophysiological aspects, and several theories have been proposed to explain its mechanisms all intricately linked to one another and collectively contributing to the complex aging process. These theories encompass various aspects of aging, with cellular senescence serving as a central element (Dimri et al., 1995; Van Deursen, 2014; Shvedova et al., 2022). Cellular senescence, is closely connected to telomere shortening as cells age (Allsopp et al., 1992; Sergiev et al., 2015), and leading to decreased proliferative capacity, exacerbated by the chronic inflammation that often accompanies aging (Howcroft et al., 2013; Nikolakis et al., 2013; Zouboulis and Makrantonaki, 2019). Mitochondrial DNA single mutations can also enhance cellular senescence (Michikawa et al., 1999), as they affect mitochondrial function, potentially leading to increased free radical production. Free radicals, in turn, can cause oxidative damage and exacerbate the aging process (Hekimi et al., 2011; Lee et al., 2021). Additionally, they can lead to the degradation of the extracellular matrix (ECM) by activating matrix metalloproteinases (Lee et al., 2021).

MicroRNAs (miRNAs) which regulate gene expression post-transcriptionally, play a dual role in aging. They can influence cellular senescence and modulate the expression of genes related to inflammation and oxidative stress, impacting various aspects of aging. Furthermore in the context of the skin, they function as biomarkers for chronological aging, photoaging, and age-related diseases (Shin et al., 2011; Kinser and Pincus, 2020). Other significant mechanisms contributing to aging involve the accumulation of Advanced Glycation End Products (AGEs). AGEs exhibit a significant correlation with cellular senescence throughout the body, as well as age related indicators (Xin et al., 2021). Resulting in the stiffening and reduced elasticity of tissues throughout the body (Farrar, 2016). They also play a role in photoaging by causing abnormal aggregation of glycated elastin fibers in the skin (Zhang and Duan, 2018). By recognizing these intricate connections among aging mechanisms, we can develop a more holistic understanding of how they collectively drive the aging process. In skin aging, the key cellular perturbation is the disruption of the oxidative balance (Rittié and Fisher, 2015; Papaccio et al., 2022). In chronological aging, reactive oxygen species (ROS) production occurs primarily through cellular oxidative metabolism during adenosine triphosphate (ATP) generation and mitochondrial dysfunction. On the other hand, extrinsic aging results from the loss of redox equilibrium due to environmental factors like UV radiation, pollution, cigarette smoking, and inadequate nutrition. During aging, oxidative stress arises from increased ROS production and decreased levels of enzymatic and non-enzymatic protectors (Papaccio et al., 2022).

### 2.2 Physiological changes in aged skin

Over time, skin aging results from a complex interplay of cellular senescence and various molecular mechanisms, significantly impacting both the skin’s physiology and appearance. After the age of thirty, both cell division in the epidermal germinative layer (McKnight et al., 2022) and melanocytes in the epidermis are impacted by cellular senescence during the skin aging process (Park et al., 2023). Furthermore, the dermis experiences an annual reduction of approximately 1% in collagen content (McKnight et al., 2022) due to factors such as diminished collagen synthesis and increased degradation of existing collagen. Senescent fibroblasts in the dermis play a pivotal role in this process by producing Matrix Metalloproteinases (MMPs) as part of the Senescence-Associated Secretory Phenotype (SASP), which is responsible for collagen degradation (Lämmermann et al., 2018). The synergistic decline in collagen synthesis and the concurrent increase in collagen degradation significantly contribute to reduced skin elasticity, the formation of wrinkles, and sagging. While collagen loss occurs in non-exposed areas as well, persistent exposure to high levels of UV radiation from sunlight can further accelerate this process and lead to a reduction in the skin’s protective functions, resulting in various acute and chronic effects. The acute effects include DNA damage, suppression of DNA synthesis, cell death, and cause erythema (skin redness). On the other hand, chronic damage includes photoaging, which is characterized by premature aging signs, and an increased risk of developing epidermal cancer (McKnight et al., 2022).

Chronologically aged and photoaged skin exhibit clinical differences, but they share similar biochemical and cellular characteristics, including ROS generation, DNA damage, and ECM deterioration (Rittié and Fisher, 2015). Intrinsic aging causes thinning, dryness, fine wrinkles, and sagging, while extrinsic aging leads to coarser wrinkles, severe elasticity loss, and dyspigmentation. Despite these clinical differences (Park, 2022; Walker, 2022), both processes involve common biochemical and biophysical dermal changes, contributing to wrinkles and reduced skin elasticity (Shin et al., 2023).

## 3 Management of skin aging: spotlight on physiological approaches

### 3.1 Healthy lifestyle habits

Lifestyle significantly influences the manifestation and progression of cutaneous aging. To mitigate the signs of aging and maintain skin health, it is essential to adopt specific measures. These include avoiding prolonged and unprotected sun exposure, minimizing exposure to pollutants and polluted environments, managing temperature stresses, and maintaining a healthy diet (Bonté et al., 2019).

#### 3.1.1 Nutritional skin care

The significance of nutrition for skin health has been increasingly recognized. Extensive research has highlighted the impact of an individual’s diet on aging skin and its biochemical changes (Katta et al., 2020). A balanced diet and caloric restriction (CR) are essential for healthy aging (Zia et al., 2021). Plant-based components like carotenoids, flavonoids, and vitamins, exhibit antioxidant potentials, helping to prevent and treat ROS-associated chronic conditions, and support skin health, and overall wellbeing (Kasote et al., 2015). The primary focus is on limiting or eliminating animal products (Fam et al., 2022), and some researchers may also choose to exclude or restrict processed foods within this definition. Notably, recent research has provided robust evidence supporting the beneficial impact of plant-based diets on inflammatory skin diseases (Flores-Balderas et al., 2023). These diets offer benefits by regulating inflammatory and oxidative processes, which are key mechanisms in inflammatory skin diseases (Solway et al., 2020). In the context of a plant-based diet, the consumption of a wide variety of plant-based functional foods, including nutrient-rich options like mangos (Dattola et al., 2020; Fam et al., 2020), almonds, and avocados, have been associated with observable positive effects on skin health. The regular and appropriate intake of these foods may contribute to improved condition of the skin, owing to their essential vitamins, minerals, antioxidants, and healthy fats, potentially aiding in the management of inflammatory skin diseases (Dattola et al., 2020; Fam et al., 2022). Mango extract was shown to reduce UVB-induced wrinkles and demonstrated anti-aging effects in mice, suggesting potential benefits for humans (Song et al., 2013). One clinical trial found that consuming a moderate amount of Ataulfo mango had a positive effect on reducing facial wrinkle depth and severity. However, higher quantities of mango intake were associated with an increase in facial wrinkles (Fam et al., 2020). Almond intake was found to significantly improve facial wrinkles (Foolad et al., 2019; Rybak et al., 2021), and it decreased the intensity of facial pigmentation without adverse effects (Rybak et al., 2021). The antioxidant properties of alpha tocopherol (vitamin E) in almonds may contribute to these beneficial effects, potentially preventing or reducing the progression of wrinkles and facial pigmentation, thus aiding in the prevention of skin aging (Flores-Balderas et al., 2023). Almond oil and almond extract, applied topically in animals (Sultana et al., 2007) and *in vitro* studies (Evans-Johnson et al., 2013), showed promise in reducing the histologic damage associated with skin photoaging. However, further human clinical trials are necessary to validate their effectiveness and safety. Several studies have shown that avocados possess beneficial properties due to compounds like carotenoids, monounsaturated fatty acids, and phenols. While they have long been recognized for their positive effects on skin elasticity when applied topically, studies have revealed that consuming avocados also provides skincare benefits (Dreher and Davenport, 2013). Oral consumption of avocados has been shown to positively impact skin health, leading to enhanced skin elasticity and firmness (Henning et al., 2022). AGE formation is a significant mechanism of skin aging and exerts pathophysiological effects through both endogenous (dietary sugar) and exogenous (cooking) routes. Endogenous AGEs are formed when sugar in the diet binds covalently with donor molecules. Exogenous AGEs are the result of cooking processes, with grilling, frying, and roasting producing higher levels compared to boiling and steaming. Interestingly, certain dietary compounds have shown potential as inhibitors of glycation-mediated aging, offering promising avenues for further research and intervention (Nguyen and Katta, 2015). Reducing sugar intake and improving glycemic control in patients with diabetes are effective measures to decrease skin collagen glycation and its associated consequences, ultimately helping to prevent premature skin aging (Lyons et al., 1991). Emerging evidence has suggested that a balanced diet with functional components like phytochemicals (Tong et al., 2019), functional proteins (Figueres Juher and Basés Pérez, 2015), peptides (Song et al., 2017), functional sugars (Wen et al., 2016), functional oils (Shahbakhti et al., 2004; Romana-Souza and Monte-Alto-Costa, 2019), probiotics (Kimoto-Nira, 2018; Im et al., 2019), vitamins (Mukherjee et al., 2006; Bagatin et al., 2014; Keen and Hassan, 2016; Pullar et al., 2017), and minerals (Driscoll et al., 2010; Choi et al., 2018; Pickart and Margolina, 2018) has been shown to improve the appearance and function of skin affected by photoaging. Nutrients like vitamins C and E, zinc, and omega-3 fatty acids are known to promote healthy skin and may aid in reducing the appearance of wrinkles (Schagen et al., 2012). Antioxidant-rich food supplements can help to prevent and treat conditions associated with ROS, reducing oxidative stress and promoting a healthier, longer lifespan. However, a balanced diet remains essential, and it is advisable to consult healthcare professionals before initiating any supplement regimen (Liu et al., 2018).

Other promising interventions for health and longevity, like CR and intermittent fasting, have shown potential effects on the skin, although their impact is not yet fully understood. The study by Cefalu in 1995 demonstrated that CR can reduce skin protein glycation in rats, resulting in a decrease in the accumulation of AGEs in collagen.(Cefalu et al., 1995).

In addition, studies have shown that mice on a CR diet experienced reduced skin irritation induced by retinoids (Varani et al., 2008). Moreover in a recent study mice subjected to 30% CR before UVB irradiation ed exhibited notable protection against skin damage and inflammation (Tang et al., 2022).

#### 3.1.2 Physical activity

Physical activity has been linked to numerous health advantages, such as improved cardiovascular fitness, weight management, and enhanced mental wellbeing. In addition to these benefits, emerging research has suggested that maintaining an active lifestyle could also have a positive impact on skin health (Skroza et al., 2020). Furthermore, exercise has been shown to play a valuable role in enhancing wound healing in aged skin. This has been demonstrated through studies conducted on both mice (Keylock et al., 2008) and humans (Emery et al., 2005). However, despite these positive findings, the specific effects of exercise on skin aging are still not fully understood. One study demonstrated the beneficial effects of endurance exercise on skin aging, revealing exercise-induced IL-15 as a novel regulator of mitochondrial function in aging skin (Crane et al., 2015). Furthermore, an extremely recent study reported that resistance training rejuvenates aging skin by both reducing circulating inflammatory factors and enhancing dermal extracellular matrices (Nishikori et al., 2023).

### 3.2 Chronotherapy

Increasing understanding of circadian rhythms has led to the development of chronotherapy in clinical practice. The primary objective of chronotherapy is to enhance the tolerability and effectiveness of treatments by synchronizing drug administration with the natural oscillations of drug and treatment targets in diseased tissues, thereby minimizing toxicity and side effects (Dong et al., 2020). Circadian medicine and chronotherapy are gaining prominence as vital aspects of preventive medicine and patient care (Sulli et al., 2018; Jacob et al., 2020; Deota and Panda, 2021). This approach involves considering circadian rhythms during pre-clinical research and disease management planning, with the ultimate goal of improving clinical outcomes while also minimizing the occurrence of side effects. Skin exhibits circadian variations in various functions, regulated by circadian clock genes (Zanello et al., 2000)., For instance, the outermost layer of the skin, known as the stratum corneum, displays rhythmic changes, exhibiting higher permeability in the evening compared to the morning (Yosipovitch et al., 1998). This permeability is influenced by Aquaporin 3 (AQP3) in the epidermis, which is regulated by a molecular clock and plays a significant role in transepidermal water loss (Matsunaga et al., 2014). The clinical significance of circadian patterns in the skin suggests that applying moisturizers and topical treatments during the evening hours may provide increased benefits. Moreover, skin blood flow follows a circadian rhythm, peaking in the late afternoon and at night, this can impact the effectiveness of topical medications and their absorption (Smolander et al., 1993). A recent study investigated the effects of a circadian-based dual serum system LUMIVIVE System (LVS) on individuals with moderate-severe photodamage. The active group received LVS (daytime serum and nighttime serum) along with basic skin care (moisturizer and sun protection factor (SPF) 35 sunscreen), while the control group received only basic skin care. The active group showed significant improvements in radiance, overall photodamage, tactile toughness, and fine lines. These positive results were supported by various assessments, including clinical grading, subject self-assessment, standardized photography, punch biopsies, and skin surface oxidation analysis (Draelos et al., 2020). Indeed, the application of topical anti-aging skincare products incorporating chronotherapy is recognized for its effectiveness in promoting graceful aging.

### 3.3 Endogenous antioxidant molecule: focusing on melatonin

The skin produces various protective molecules such as melatonin, vitamin D, and melanin. In addition to their diverse functional properties, these molecules also possess unique antioxidant properties to counteract oxidative stress. (Brenner and Hearing, 2008; Slominski et al., 2014; Slominski et al., 2018; Bikle and Christakos, 2020). Active metabolites of vitamin D3 (D3) play a vital role in promoting skin health. These metabolites are known to exert their beneficial effects through various mechanisms, including prevention of DNA damage, and the stimulation of DNA repair mechanisms, immunomodulation, anti-inflammatory actions, regulation of keratinocyte proliferation and particularly potent antioxidative responses, (Bocheva et al., 2021). Traditionally it has long been accepted that skin color plays a crucial role in protecting the body from the harmful effects of sunlight. This is mainly because melanin, the pigment responsible for skin color, not only absorbs a wide range of UV radiation but also acts as a powerful antioxidant (Brenner and Hearing, 2008). As the skin ages, its endogenous antioxidant capacity decreases, resulting in the accumulation of oxidative damage. Melatonin, a hormone primarily released by the pineal gland, plays crucial roles in regulating circadian rhythms and promoting sleep (Reiter et al., 2010). Notably, it is synthesized within human skin, serving as a versatile molecule (Slominski et al., 2018). Among the numerous endogenous antioxidant molecules, melatonin distinguishes itself as a prominent contributor to combating oxidative damage. Skin-produced melatonin provides protective effects against external damage (Janjetovic et al., 2014), reducing cutaneous harm by scavenging toxic radicals and inhibiting their generation, especially at the mitochondrial level (Fischer et al., 2008a; Galano, 2011; Reiter et al., 2018). Melatonin’s higher concentration in mitochondria enables it to preserve optimal mitochondrial function, countering the aging process (Cedikova et al., 2016). As a result, melatonin plays a crucial role in regulating mitochondrial processes (Reiter et al., 2008; Mocayar Marón et al., 2020). Additionally, melatonin stimulates antioxidant enzyme production, helps repair DNA damage, and exhibits anti-inflammatory and anti-apoptotic effects (Rodriguez et al., 2004; Fischer et al., 2008a; Fischer et al., 2008b; Park et al., 2018). As the skin ages, its natural melatonin production diminishes. To counter this decline, the topical application of melatonin presents a promising approach to elevate endogenous melatonin levels. This promising strategy has been effective in providing photoprotection and serves as a potential anti-aging approach (Milani and Sparavigna, 2018; Bocheva et al., 2022b). Numerous studies have consistently shown that melatonin has significant clinical potential as a broad-range photoprotector (Kleszczyński et al., 2011; Skobowiat et al., 2018). A recent research showed that melatonin partially inhibits premature senescence in primary melanocytes induced by UVB. This protection is achieved by reducing p53 activity and inhibiting melanin production through the downregulation of tyrosinase (TYR) expression. This highlights melatonin’s role in inhibiting senescence-associated pigmentation by acting through the p53-TYR pathway (Ma et al., 2023). Recent studies have indicated that skin care products containing melatonin can be highly effective in preventing premature skin aging caused by pollutants, heavy metals, and cigarette smoke (Sagan et al., 2017; Granger et al., 2020; Bielach-Bazyluk et al., 2021).

### 3.4 Natural topical anti-aging skin care

Consumers nowadays prioritize their health and are actively seeking cosmetics and products with natural bioactive or functional ingredients. They believe that incorporating these ingredients can make the products healthier, matching their preference for health-conscious choices (Aguiar et al., 2016; Wen et al., 2017). A good natural topical anti-aging skin care product strengthens the skin’s barrier function, provides antioxidant properties, encourages epidermal rejuvenation and/or thickening, tackles age-related pigmentation, and boosts connective tissue production (Ahmed et al., 2020). As the skin is a highly complex system, ongoing research focuses on developing advanced delivery systems and natural nanocompounds to transport high-performance ingredients deep into the dermis for superior skincare results (Hameed et al., 2019; Sharma et al., 2022). For instance, phenolic compounds from natural products are vulnerable to environmental factors like heat, light, oxidants, and metal ions during processing and storage, which can diminish their biological efficiency and resilience. Nanoencapsulation technology offers promise in tackling these issues (Liu et al., 2019). Nanoencapsulation shields sensitive compounds from external factors, optimizing their effectiveness and stability. This leads to more potent and long-lasting skincare benefits. Furthermore, as previously discussed, the application of chronotherapy can help overcome the penetration limitations of topical agents, enabling improved delivery of beneficial compounds during specific times when the skin’s receptivity is at its peak.

#### 3.4.1 Polyphenols

Polyphenols are found in various plant-based foods, including fruits, vegetables, tea, wine, cocoa, aromatic plants, and coniferous bark, with over 8,000 identified structures (Tsao, 2010). Various polyphenols exhibit significant health-promoting effects due to their potent antioxidant properties. Moreover, they possess anti-inflammatory, anti-aging, antimicrobial, and photoprotective properties (De Lima Cherubim et al., 2020).

Experimental studies have revealed that the topical application of polyphenols can effectively combat skin changes caused by both photoaging and chronological aging. These polyphenols help to restore keratinocyte structure, stimulate collagen synthesis, enhance vascularization, and normalize hyper-keratinization, making them beneficial in various stages of the skin aging process. Phenolic acids also have anti-inflammatory and immune-boosting properties, prevent collagen breakdown, and improve blood circulation, leading to significant anti-aging benefits (Luo et al., 2021). Furthermore, one study suggested that polyphenols can modulate age-related signaling pathways in dermal fibroblasts, vital cells responsible for collagen production and skin elasticity (Lee et al., 2022). Polyphenolic compounds like resveratrol, proanthocyanins, and silymarin have been studied for their impact on animal models exposed to DNA damage, oxidative stress, and UV-induced skin irritation (Dunaway et al., 2018). Particularly, resveratrol has garnered significant attention over the last 2 decades and is considered as a promising anti-aging ingredient with potential skin benefits (Camins et al., 2009). A study involving HaCat cells exposed to the nitric oxide free radical donor sodium nitroprusside demonstrated that resveratrol provides protection for human skin (Bastianetto et al., 2010). In another *in vitro* study, resveratrol was found to increase cell proliferation in a dose-dependent manner and significantly inhibit collagenase activity (Giardina et al., 2010).

#### 3.4.2 Natural UV- absorption compounds for sun protection

Natural antioxidants and sun blockers have gained attention for their potential as sunscreen ingredients. While their exact sunscreen mechanism is not fully understood, they are believed to protect genetic material from UV damage through the conjugated π system. This system comprises a chain of alternating single and multiple bonds that enable electron movement within molecules. This property enhances stability, UV absorption, and antioxidant capabilities, which are crucial for the effectiveness of certain natural UV filters (Cockell, 1998). Natural compounds, especially botanical UV filters with aromatic rings, offer promising potential as UV filters, surpassing synthetic products in both UV absorption and antioxidant capacities. Although they do have limitations, such as lower specific extinction values and difficulties in large-scale cosmetic applications compared to artificial sunscreens. However, they have successfully reduced the overall dependency on conventional UV filters, offering a more sustainable alternative for sun protection (He et al., 2021). Botanical UV filters are particularly noteworthy, as they offer a broad absorption spectrum at wavelengths from 200 to 400 nm and possess strong antioxidant properties (Li et al., 2023). UV sunscreens derived from natural products should possess essential features. First, they must efficiently absorb UV radiation, providing broad-spectrum protection covering UVA and UVB rays. Second, their stability in the presence of UV light is crucial to ensure that their protective efficacy remains intact and undiminished (Smaoui et al., 2017; Yarovaya et al., 2022). Furthermore, desirable natural sunscreen compounds should exhibit high efficacy at low concentrations for practical formulation and must be safe, with minimal systemic absorption (Mancebo et al., 2014).

Flavonoids, especially rutin and quercetin, have been extensively studied for their photoprotective properties, owing to their effective UV absorption abilities (Baldisserotto et al., 2018; Catelan et al., 2019; Vijayakumar et al., 2020; Mejía-Giraldo et al., 2022), but their SPF values are generally modest, except for the extract from *Hylocereus polyrhizus*, which shows remarkable photoprotective effects (Vijayakumar et al., 2020). A recent study analyzing extracts from the marine sponge *Clathria faviformis* using a molecular networking led to the discovery of a novel family of potential UV-protecting phospholipids known as favilipids (Scarpato et al., 2023). This finding presents exciting possibilities for their potential use as sunscreen ingredients or in other photoprotective applications. In addition to favilipids, certain polysaccharides have shown promising photoprotective properties. For instance, the polysaccharide from *Sophora japonica* L. flower buds protects against UV damage by reducing ROS and scavenging free radicals (Li et al., 2019). White garlic fructan has also shown its ability to protect against UVB-induced keratinocyte damage (Chen et al., 2013). Lignin is also gaining attention as a natural UV protection ingredient in sunscreens. When incorporated with other materials, it enhances UV protection capabilities. However, using lignin as a UV blocker presents challenges due to its complex structure, brownish color, and impurities (Sadeghifar and Ragauskas, 2020). Some researchers have developed methods to purify lignin, making it a valuable addition to sunscreens (Lee et al., 2019). Furthermore, Mycosporine-like amino acids (MAAs) are natural UV-absorbing compounds that can disperse absorbed energy as heat without generating ROS, making them potential alternatives to chemical sunscreens (Kageyama and Waditee-Sirisattha, 2019). Utilizing the pharmacological properties and UV protective abilities of MAAs could lead to the production of eco-friendly sunscreens. However, challenges persist, such as stimulating MAA biosynthesis for higher yields and targeted UVA and UVB absorption. More research is required to achieve controlled MAA production and optimize its incorporation into sunscreen formulations for improved UV protection (Rosic et al., 2023).

### 3.5 Regenerative medicine

Skin regeneration methods that employ endogenous therapies are safe and effective, as they align closely with the natural physiology of the body. These treatments harness the body’s own regenerative potential by using endogenous stem cells like mesenchymal stem cells (MSCs) and tissue-induced pluripotent stem cells (iPSCs), or by utilizing extracellular vesicles/exosomes derived from endogenous MSCs. In contrast to conventional treatments for facial wrinkles, like dermal fillers, injectables, microneedle, fractional laser, and cosmeceuticals, which often have limited efficacy and safety, regenerative medicine-based skin therapies offer a more promising solution (Ntege et al., 2020). These therapies stimulate multi-directional differentiation, self-replication, immuno-modulation, inflammation regulation, angiogenesis, and hemostasis, closely mirroring the skin’s natural regenerative mechanisms (Shimizu et al., 2022). MSCs indeed hold great promise for skin regeneration and wound healing due to their remarkable characteristics, including immune tolerance, angiogenesis stimulation, chemo-attraction to damaged tissue, and inflammation modulation (Jo et al., 2021). However, despite these advantages, regenerative therapies face challenges related to tissue origin, donor factors, isolation procedures, potential adverse effects, and ethical regulations (Shimizu et al., 2022). Overcoming these challenges is crucial to fully harness the potential of advancing regenerative medicine for improved skin health.

#### 3.5.1 Adipose-derived mesenchymal stem cells

Adipose-derived mesenchymal stem cells (AD-MSCs) are favored for skin anti-aging treatment due to their efficiency in re-epithelialization and secretion of different growth factors. Their abundance, easy isolation, and capacity to enhance angiogenesis and synthesize dermal elastic matrix components make them valuable for skin regeneration (Meruane et al., 2012; Gaur et al., 2017). Experimental studies support the use of AD-MSCs to improve the quality of aging skin (Charles-de-Sá et al., 2015) and emphasize the functional benefits of ADSCs, such as glycation suppression, antioxidation, and trophic effects (Zhang et al., 2014). Additionally, one study revealed that protein extracts from ADSCs medium exhibited anti-aging and whitening effects via microneedles on Asian skin (Wang et al., 2018). Moreover, AD-MSCs were found to protect against oxidative stress by promoting human dermal fibroblast proliferation and inhibiting ROS production through the secretion of various cytokines (Gentile and Garcovich, 2021).

#### 3.5.2 Cell-free therapy

To promote healthy skin and combat signs of aging, regenerative cell-free approaches encompass autologous techniques like platelet-rich plasma (PRP) and autologous conditioned serum (ACS), also known as blood cell secretome (BCS). BCS involves enriching blood serum with bioactive secretions from blood cells, essential for clotting, infection prevention, wound healing, and tissue restoration. These techniques typically entail the preparation and local application of these substances to enhance skin rejuvenation and healing (Kerscher et al., 2022). BCS has demonstrated effectiveness in treating musculoskeletal disorders. It contains inflammation-resolving cytokines, growth factors, exosomes, and lipid mediators. Pre-clinical data have suggested that BCS may also offer potential benefits in combating skin aging. Clinical study conducted by Kerscher et al., in 2021, demonstrated that BCS significantly improves skin firmness and hydration after 12 and 24 weeks of use (Kerscher et al., 2021). In addition to the clinical evidence, *in vitro* experiments conducted on a well-described skin model further support BCS as a beneficial agent for cell division and overall skin quality (Schmitt et al., 2018). On the other hand, PRP is a widely used autologous procedure for various treatments, including skin rejuvenation. It contains leukocytes with catabolic and pro-inflammatory activity, balanced with plasma and growth factors that have anabolic functions for tissue healing and growth (Buzalaf and Levy, 2022). Several studies on PRP monotherapy have shown increased fibroblast numbers, denser dermal collagen and elastic fibers, thicker dermal-epidermal junction, and increased epidermal thickness, indicating its potential for tissue regeneration and skin rejuvenation (Abuaf et al., 2016; Cabrera-Ramírez et al., 2017; Charles-de-Sá et al., 2018). The utilization of platelet concentrates has garnered considerable interest, yet its clinical efficacy is controversial primarily due to the inherent variability in composition observed among individual patients.(Bajaj et al., 2022; Kerscher et al., 2022). To address this issue, ongoing efforts aim to define optimized protocols for using PRP in skin treatments, ensuring more reliable and predictable outcomes (Chahla et al., 2017; Bansal et al., 2021). Additionally second- and third-generation platelet concentrates are platelet-rich fibrin (PRF) and injectable platelet-rich fibrin (i-PRF) have emerged. PRF is obtained with a single centrifugation step and no anticoagulants are needed. Both are safe and well-tolerated for skin rejuvenation (Buzalaf and Levy, 2022). However, PRP has more extensive literature with favorable results (Maisel-Campbell et al., 2020), but with low study quality, requiring further research. PRF and i-PRF are easier to obtain and show promise for increased collagen production *in vitro*, especially under lower relative centrifugation force (RCF). Nevertheless, there are limited clinical trials evaluating the effectiveness of PRF and i-PRF for skin rejuvenation. More research is needed to fully understand their potential benefits and clinical applications (Buzalaf and Levy, 2022). Table1 provides a summary of various approaches discussed in the review article. The approaches are organized into categories that include their interventions, mechanisms of action, and the benefits they offer in the prevention of skin aging, along with corresponding references.

**TABLE 1:** Summary of various approaches discussed in the review article. Abbreviations: ROS, reactive oxygen species; CR, caloric restriction; AQP3, Aquaporin 3.

	Mechanism of action	Benefits	References
Healthy lifestyle habits	• Counters ROS	• Improved skin condition	Katta et al. (2020),Zia et al. (2021), Kasote et al. (2015), Flores-Balderas et al. (2023), Solway et al. (2020),Dattola et al. (2020), Fam et al. (2020), Foolad et al. (2019), Rybak et al. (2021), Evans-Johnson et al. (2013), Henning et al. (2022), Nguyen and Katta (2015), Lyons et al. (1991), Tong et al. (2019), Figueres Juher and Basés Pérez (2015), Song et al. (2017), Wen et al. (2016), Shahbakhti et al. (2004), Romana-Souza and Monte-Alto-Costa (2019), Kimoto-Nira (2018), Im et al. (2019), Mukherjee et al. (2006), Bagatin et al. (2014), Keen and Hassan (2016), Pullar et al. (2017), Driscoll et al. (2010), Choi et al. (2018), Pickart and Margolina (2018), Schagen et al. (2012), Liu et al. (2018), Cefalu et al. (1995), Varani et al. (2008), Tang et al. (2022). Skroza et al. (2020), Keylock et al. (2008), Emery et al. (2005), Crane et al. (2015), Nishikori et al. (2023)
• Antioxidant-rich diet	• Regulates inflammation	• Reduced inflammation	
• Plant-based diet	• Provide essential nutrients	• Potential wrinkle reduction	
• Nutrient-rich foods	• Reduces glycation and AGE accumulation	• Enhanced elasticity	
• CR	• Reduced skin irritation induced by retinoids	• Protection against damage and inflammation	
• Reducing sugar intake	• Decreases collagen glycation	• Helps overcome penetration limitations of topical agents	
• Regular physical activity	• Reducing inflammatory factor	• Accelerated wound healing	
• Cardio workout	• Regulation of mitochondrial function	• Potential rejuvenation of aging skin	
• Endurance exercises	• Enhancing dermal extracellular matrices		
Chronotherapy	• Synchronizing drug administration with circadian rhythm of skin blood flow	• Enhanced treatment effectiveness	Dong et al. (2020), Sulli et al. (2018), Jacob et al. (2020), Deota and Panda (2021)
		• Improved tolerability of treatments	
• Aims to improve clinical outcomes while minimizing side effects	• Timing for the application of skincare products align with skin permeability (Influence of AQP3)	• Minimized toxicity and side effects	
		• Demonstrated improvements in radiance od skin and photodamage	
Endogenous Antioxidant Molecule	• Mitochondrial Protection	• Preventing premature skin aging caused by pollutants, heavy metals, and cigarette smoke	Brenner and Hearing (2008), Slominski et al. (2014), Slominski et al. (2018), Bikle and Christakos (2020), Bocheva et al. (2021), Janjetovic et al. (2014), Galano (2011), Reiter et al. (2010), Cedikova et al. (2016), Mocayar Marón et al. (2020), Rodriguez et al. (2004), Fischer et al. (2008a), Fischer et al. (2008b), Park et al. (2018), Milani and Sparavigna (2018), Bocheva et al. (2022b), Kleszczyński et al. (2011), Skobowiat et al. (2018), Ma et al. (2023), Sagan et al. (2017), Granger et al. (2020), Bielach-Bazyluk et al. (2021)
	• Regulation of Mitochondrial Processes		
• Role of Melatonin in Skin Protection	• Stimulation of Antioxidant Enzymes and DNA Repair	• providing photoprotection and potential anti-aging effects	
	• Exhibits anti-inflammatory and anti-apoptotic effects		
Natural Topical Anti-Aging Skin care	• Optimizing effectiveness and stability	• Strengthen the skin’s barrier function	Ahmed et al. (2020), Hameed et al. (2019), Sharma et al. (2022), Tsao (2010), Luo et al. (2021), Tsao. (2010), De Lima Cherubim et al. (2020), Luo et al. (2021),Lee et al. (2022), Dunaway et al. (2018),Camins et al. (2009), Bastianetto et al. (2010),Giardina et al. (2010), Giardina et al. (2010),He et al. (2021), Li et al. (2019, 2023), Smaoui et al. (2017), Yarovaya et al. (2022), Baldisserotto et al. (2018), Catelan et al. (2019), Vijayakumar et al. (2020), Mejía-Giraldo et al. (2022), Scarpato et al. (2023), Chen et al. (2013), Sadeghifar and Ragauskas (2020), Lee et al. (2019), Kageyama and Waditee-Sirisattha (2019), Rosic et al. (2023)
• Natural bioactive or functional ingredients	• Shields sensitive compounds from external factors	• Encourage epidermal rejuvenation	
• Focuses on advanced delivery systems	• Potent antioxidant properties	• More potent and long-lasting skincare	
• Polyphenols	• Anti-inflammatory, anti-aging, antimicrobial, and photoprotective properties		
• Natural UV- absorption compounds for sun protection	• Modulate age-related signaling pathways in dermal fibroblasts		
	• Increases cell proliferation while inhibiting collagenase activity		
	• Improve blood circulation		
Regenerative Medicine	• Re-epithelialization and secretion of various growth factors	• More effective in treatment of facial skin aging compared to conventional treatments	Ntege et al. (2020), Shimizu et al. (2022), Jo et al. (2021) Meruane et al. (2012),Gaur et al. (2017),Zhang et al. (2014),Wang et al. (2018), Gentile and Garcovich (2021), Kerscher et al. (2021,2022),Schmitt et al. (2018), Buzalaf and Levy (2022),Abuaf et al. (2016), Cabrera-Ramírez et al. (2017),Charles-de-Sá et al. (2018),Bajaj et al. (2022),Chahla et al. (2017),Bansal et al. (2021),Maisel-Campbell et al. (2020)
• Endogenous stem cells	• Glycation suppression	• Contains growth factors for tissue healing and growth	
• Cell free therapy	• Inhibiting ROS production through the secretion of various cytokines	• Mirroring the skin’s natural regenerative mechanisms	
• Challenges in regenerative therapies include issues related to tissue origin, isolation procedures, potential adverse effects, and ethical regulations	• Promoting human dermal fibroblast proliferation	• Easily isolated	
	• Immuno-modulation, inflammation regulation, angiogenesis	• Improving skin firmness and hydration	

Abbreviations: ROS, reactive oxygen species; CR, caloric restriction; AQP3, Aquaporin 3.

## 4 Conclusion

Skin aging is influenced by both extrinsic and intrinsic factors. While intrinsic factors are determined by genetics and the natural aging processes, and are beyond our direct control, extrinsic factors can be actively managed and modified by external interventions. The focus of this review has focused on exploring safer alternatives for managing skin aging. Strategies such as adopting a proper diet, maintaining healthy lifestyle habits, utilizing endogenous and natural compounds as antioxidants and sun protection, implementing regenerative medicine and chronotherapy in topical treatments have been emphasized. By considering these strategies, individuals may potentially promote better skin health, mitigate the impact of extrinsic aging factors, and embrace a more holistic and sustainable approach to combat skin aging. Further research and exploration in this direction may lay the foundation for safer and more effective interventions in the field of skin aging.
